# Clinical Insights of Antibioma in the Orofacial Region: A Report of Two Cases

**DOI:** 10.7759/cureus.115190

**Published:** 2026-08-25

**Authors:** Ramachandra Reddy Gowda Venkatesha, Nandhini Chandran, Karthik Rajaram Mohan, R T Reethika Rathan

**Affiliations:** 1 Oral Medicine and Radiology, Vinayaka Mission's Sankarachariyar Dental College, Vinayaka Mission's Research Foundation (DU), Salem, IND

**Keywords:** antibioma, color doppler ultrasonography, decayed teeth, orofacial, orthopantomography

## Abstract

Antibiotics are medications used as therapeutic interventions to treat and prevent the spread of microorganisms in odontogenic infections. An antibioma is a sterile, chronic, tough, thick-walled abscess that usually develops following an injudicious, excessive, repeated, or prolonged course of antibiotic therapy or inadequate surgical drainage of an infection. Clinically, an antibioma may present as a painful or painless orofacial swelling and may be associated with constitutional symptoms such as intermittent fever. We present two cases of antibioma in a 70-year-old female and a 57-year-old male, both of whom developed persistent orofacial swellings following previous antimicrobial therapy for odontogenic infections. These cases highlight antibioma as a critical iatrogenic complication of antibiotic misuse in dental practice and underscore the importance of antibiotic stewardship. Adequate surgical drainage remains the cornerstone of management of odontogenic abscesses, and clinicians should maintain a high index of suspicion for antibioma when persistent orofacial swellings fail to respond to conventional antimicrobial therapy.

## Introduction

Antibioma is a rare form of chronic, sterile inflammatory swelling characterized by the formation of a thick-walled lesion following inadequate drainage of a pre-existing abscess or inappropriate, incomplete, or prolonged use of antimicrobial therapy [1]. Unlike an acute bacterial abscess, which typically follows a predictable course of suppuration and resolution after appropriate drainage and antimicrobial treatment, an antibioma may persist as a firm, indurated mass because of organization of inflammatory exudate, fibrosis, and incomplete resolution of the underlying infection [1]. The condition may therefore present with clinical and radiological features that mimic persistent infection, neoplasia, granulomatous disease, or other chronic inflammatory disorders, making accurate diagnosis particularly challenging.

Although antibiomas have been described at several anatomical sites, the head and neck region appears to be disproportionately affected. In a previous review, it was reported that about 80.36% of antibiomas occurred in the head region; the gluteal and neck regions accounted for 10.71% and 8.92%, respectively [1]. The predominance of lesions in the head and neck may be related to the high frequency of infections in this region, repeated exposure to antimicrobial therapy, and the anatomical complexity of potential spaces that can facilitate persistence and organization of inflammatory lesions. Within the orofacial region, however, antibioma remains an uncommon and underrecognized entity, with limited documentation in the dental and oral medicine literature [2].

Orofacial antibioma represents a particularly important diagnostic challenge because its clinical manifestations are often nonspecific. Patients may present with a persistent, firm, localized swelling, variable pain or tenderness, induration, or a history suggestive of a previously treated odontogenic or soft-tissue infection. Depending on its location and duration, the lesion may present clinically as a chronic odontogenic infection, residual abscess, inflammatory pseudotumor, fibro-osseous lesion, or malignant neoplasm. Absence of overt systemic manifestations or acute inflammatory signs may further obscure the diagnosis. Furthermore, repeated empirical administration of antibiotics without any definitive source control can contribute to persistence of the lesion and further complicate the clinical course [2].

Antibioma pathogenesis results from incomplete resolution of infection with ongoing inflammatory stimulation and organization of the inflammatory exudate. Poor drainage may leave residual necrotic material and inflammatory mediators in the lesion, and inappropriate or incomplete antimicrobial therapy may suppress bacterial proliferation without eradicating the underlying nidus of infection. Subsequent recruitment of inflammatory cells, fibroblast activation, extracellular matrix deposition, and progressive fibrosis can result in the development of a well-organized, thick-walled lesion. This process may ultimately produce a clinically palpable mass that is relatively indolent and may contain little or no actively drainable pus. Consequently, the distinction between an active abscess and an antibioma is clinically significant because management strategies differ substantially [2]. 

From an oral and maxillofacial perspective, recognition of antibioma is particularly relevant because odontogenic infections are common and are frequently treated with antibiotics in routine dental practice. Antibiotics, however, should not substitute for appropriate elimination of the source of infection and adequate drainage when indicated. Failure to address the primary odontogenic focus may result in persistence or recurrence of inflammatory pathology. Moreover, the clinical picture can be modified by self-medication, early withdrawal of prescribed antibiotics, improper choice of antibiotics, and repeated courses of empirical antibiotics, which can contribute to diagnostic ambiguity. These considerations underscore the importance of detailed history taking, including documentation of prior infections, dental procedures, incision and drainage, and exposure to antibiotics [3].

Therefore, the diagnosis of an orofacial antibioma requires correlation of the clinical history with the physical examination and proper imaging findings. The clinical suspicion should be raised by a history of a previously treated infection with a persistent localized, firm swelling, especially if the lesion does not respond to antimicrobial therapy. Imaging can help to determine the anatomic extent of the lesion and its relationship to adjacent teeth, bone, fascial spaces, and soft tissue. Radiologic findings, however, may not always be pathognomonic. In selected cases, aspiration, microbiological evaluation, cytological assessment, or histopathological examination may be required to exclude alternative diagnoses and to assess the nature of the lesion [3]. Adequate eradication of persistent bacterial species from the root canal system is essential to prevent chronic inflammatory sequelae and lesion progression (Rumhein et al.) [4].

Despite its potential clinical significance, the literature regarding orofacial antibioma is scarce, and the condition can be missed or misdiagnosed as recurrent infection or other chronic inflammatory pathology. Because of the lack of well-documented cases, the clinical spectrum, the predisposing factors, the anatomic distribution, the diagnostic characteristics, and the optimal management are also poorly understood. A systematic description of individual cases can therefore contribute valuable information to clinicians who encounter persistent or atypical inflammatory swellings in the oral and maxillofacial region.

The present case series describes the clinical, radiological, diagnostic, and therapeutic characteristics of patients with orofacial antibioma. This report highlights the varied clinical presentations and diagnostic difficulties of this rare condition. This report aims to improve clinical recognition of antibioma and to emphasize the importance of thorough history taking, investigation, identification and elimination of the source of infection, and avoidance of indiscriminate use of antibiotics. Greater awareness of this entity may lead to earlier diagnosis, prevention of unnecessary repeated antimicrobial therapy, and appropriate management of persistent orofacial inflammatory lesions.

## Case presentation

Case one

**Figure 1:**
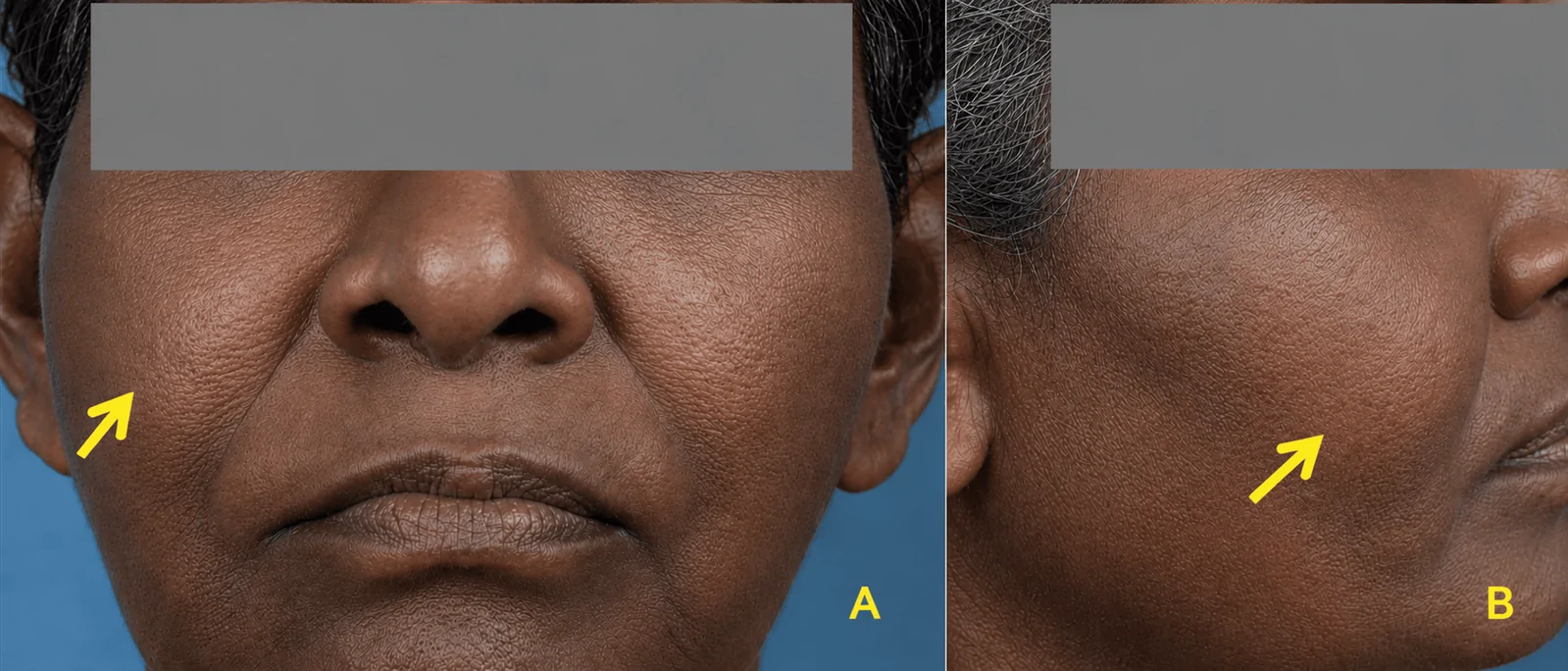
Case one frontal and right lateral profile views A) Frontal profile view showing swelling on the right middle-third of the face (yellow arrow indicates the swelling) B) Right Lateral Profile view revealed extraoral swelling in the right middle-third of the face (yellow arrow indicates the swelling).

A 70-year-old female reported to our department with a chief complaint of swelling and pain in her decayed right upper back tooth region for the past 3 days. The patient gives a history of pain and swelling in the right upper back tooth region for the past 3 days. The pain is mild, throbbing, non-radiating, aggravated by independent factors, and not relieved by antibiotic medications bought from a nearby pharmacy. The patient had no harmful oral habits, such as chewing or smoking tobacco. Her medical history revealed she was not diabetic or hypertensive. An extraoral examination revealed facial asymmetry due to swelling on the right side of the face. Extraorally, a 1.5 x 1 cm swelling is present on the right side of the middle third of the face. The extraoral swelling extends anteriorly 1.5 cm away from the right commissure of the lip, posteriorly 2.5 cm away from the right lateral canthus of the eye, superiorly 2.5 cm below the right lower eyelid, and inferiorly 4.5 cm away from the right lower border of the mandible, above and in front of the right lower border of the mandible. The skin overlying the surface of the swelling appears non-shiny and stretched. No secondary changes such as extraoral sinus, ulcer, punctum, or fistula were noted (Figures 1A, 1B).

**Figure 2:**
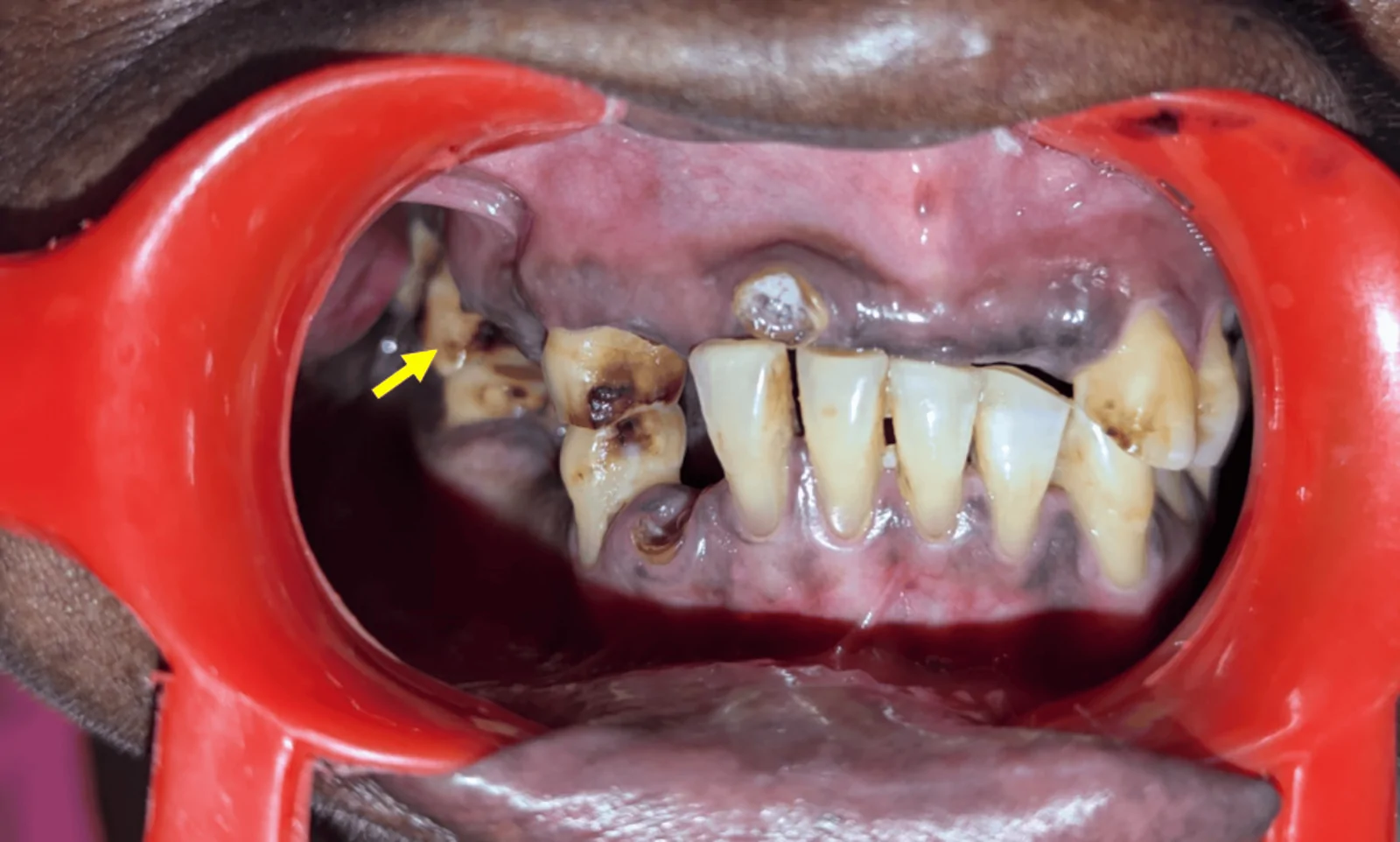
Case one - intraoral examination revealed a carious decayed right maxillary second molar (yellow arrow)

On palpation, the swelling was firm in consistency, non-tender, non-reducible, and non-compressible. Intraoral examination revealed a carious right maxillary second molar, tender on percussion (Figure 2).

**Figure 3:**
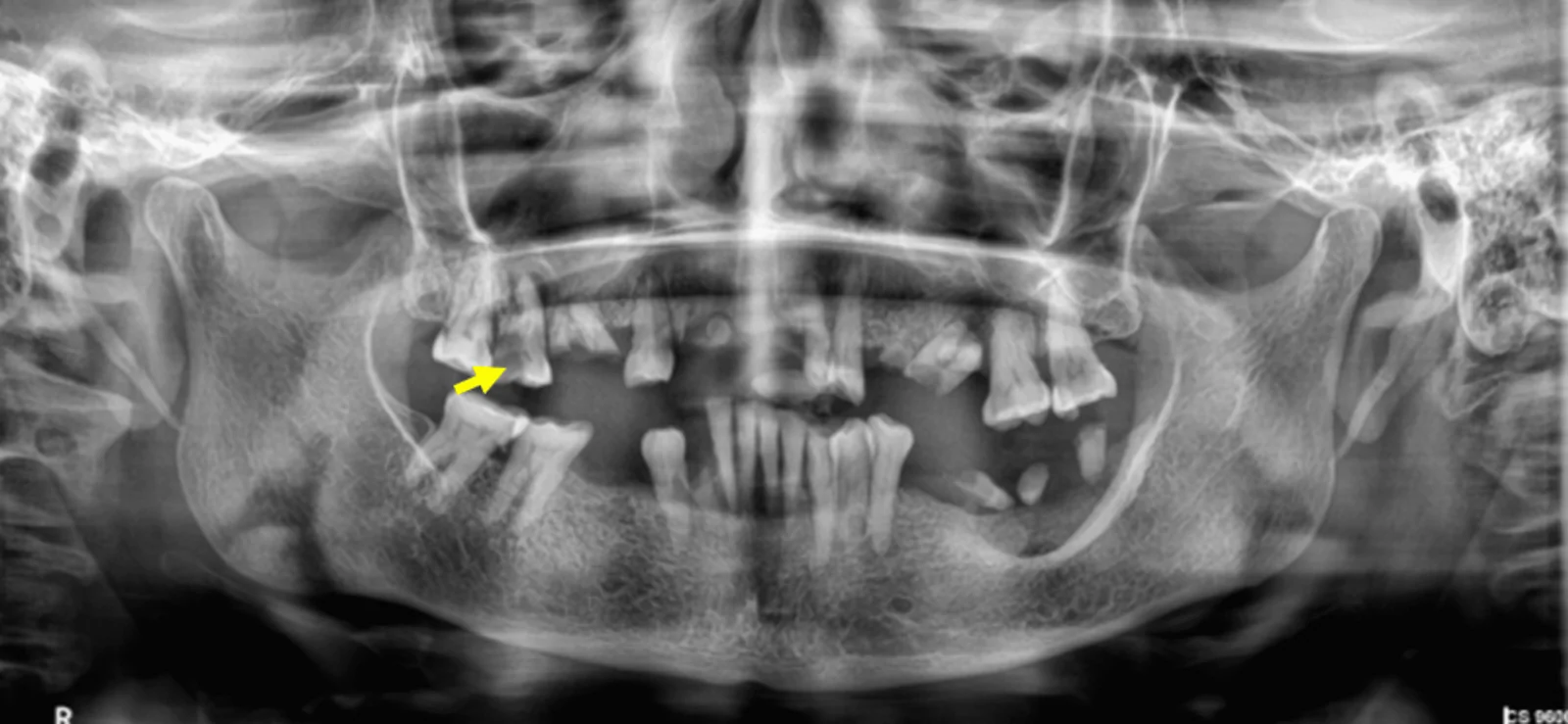
Case one - orthopantomography revealed radiolucency in the distoproximal crown portion of right maxillary second molar tooth, suggestive of distoproximal caries (yellow arrow indicates the affected carious tooth).

The orthopantomography revealed a grossly decayed carious right maxillary second molar tooth with an ill-defined radiolucency in the periapical root region with loss of lamina dura suggestive of rarefying osteitis, missing 21,24,15,36,45,46, root stumps in 11,13,16,25,26,37,38,43, dental caries in 14,28, root caries in 44, and generalized alveolar bone loss suggestive of chronic generalized periodontitis (Figure 3).

The hematological laboratory parameters are tabulated (Table 1).

**Table 1:** Case one - hematological laboratory parameters

Laboratory Parameters	Observed Value	Normal range
Random Blood Glucose	120 mg/dL	100-160 mg/dL
Haemoglobin	9.8 %	12-15 %
Bleeding time	4 minutes	3- 5 minutes
Clotting time	4 minutes	3- 8 minutes
Total White Blood Cells (WBC)	8300 cells / cu.mm	6,000-10,000 cells/ cu.mm
Neutrophils	65%	40-60%
Lymphocytes	32%	20-40%
Eosinophils	3%	1-6%
Erythrocyte Sedimentation rate	21 mm/hour	13 mm/hour

**Figure 4:**
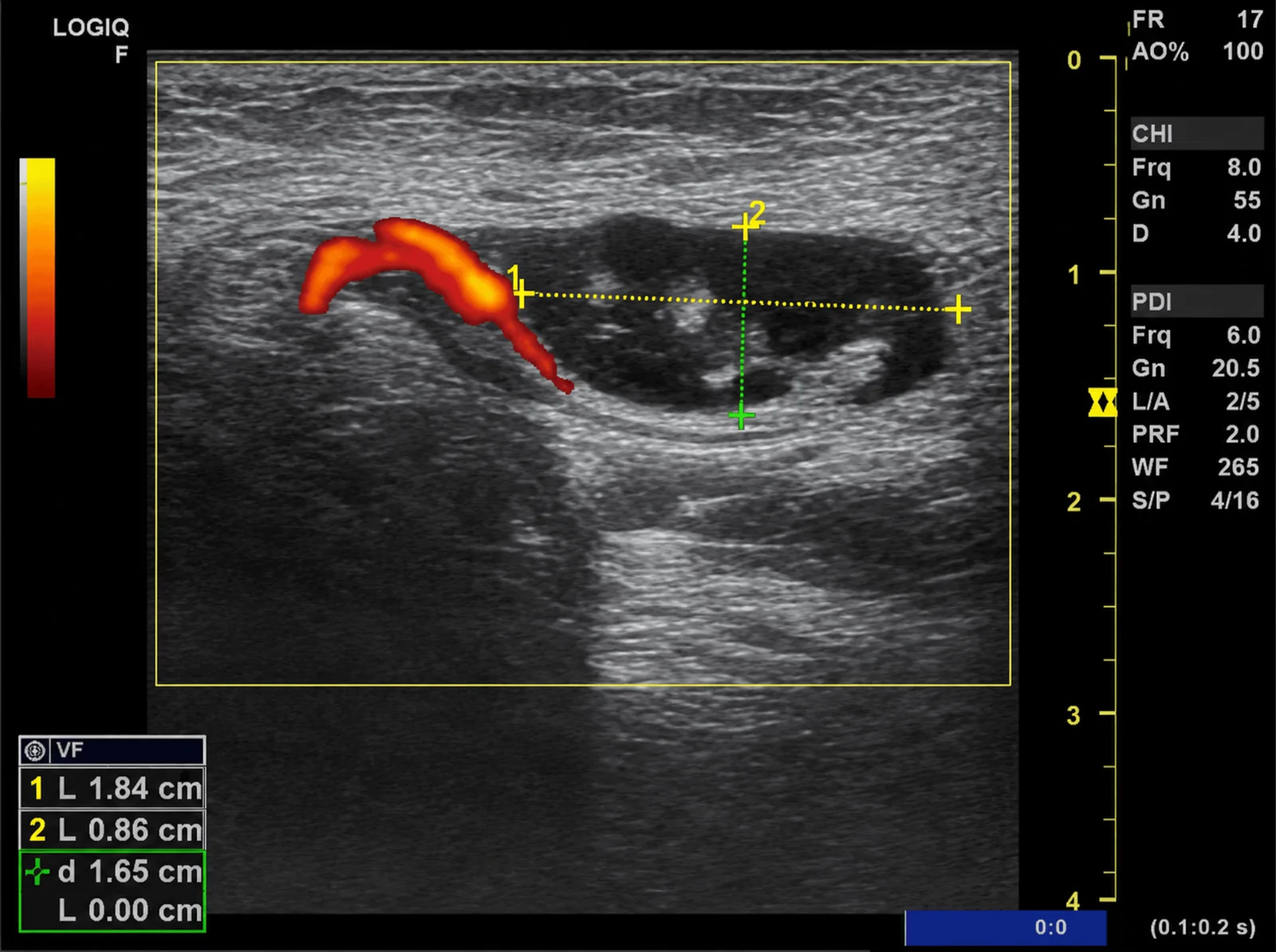
Case one - color Doppler ultrasonography revealed a well-defined hypoechoic area measuring about 1.84 cm x 1.65 cm in the right buccal space

Color Doppler ultrasonography revealed a hypoechoic area measuring 1.84 cm x 1.65 cm in the right buccal space in the subcutaneous plane with a well-defined margin (Figure 4).

Case two

**Figure 5:**
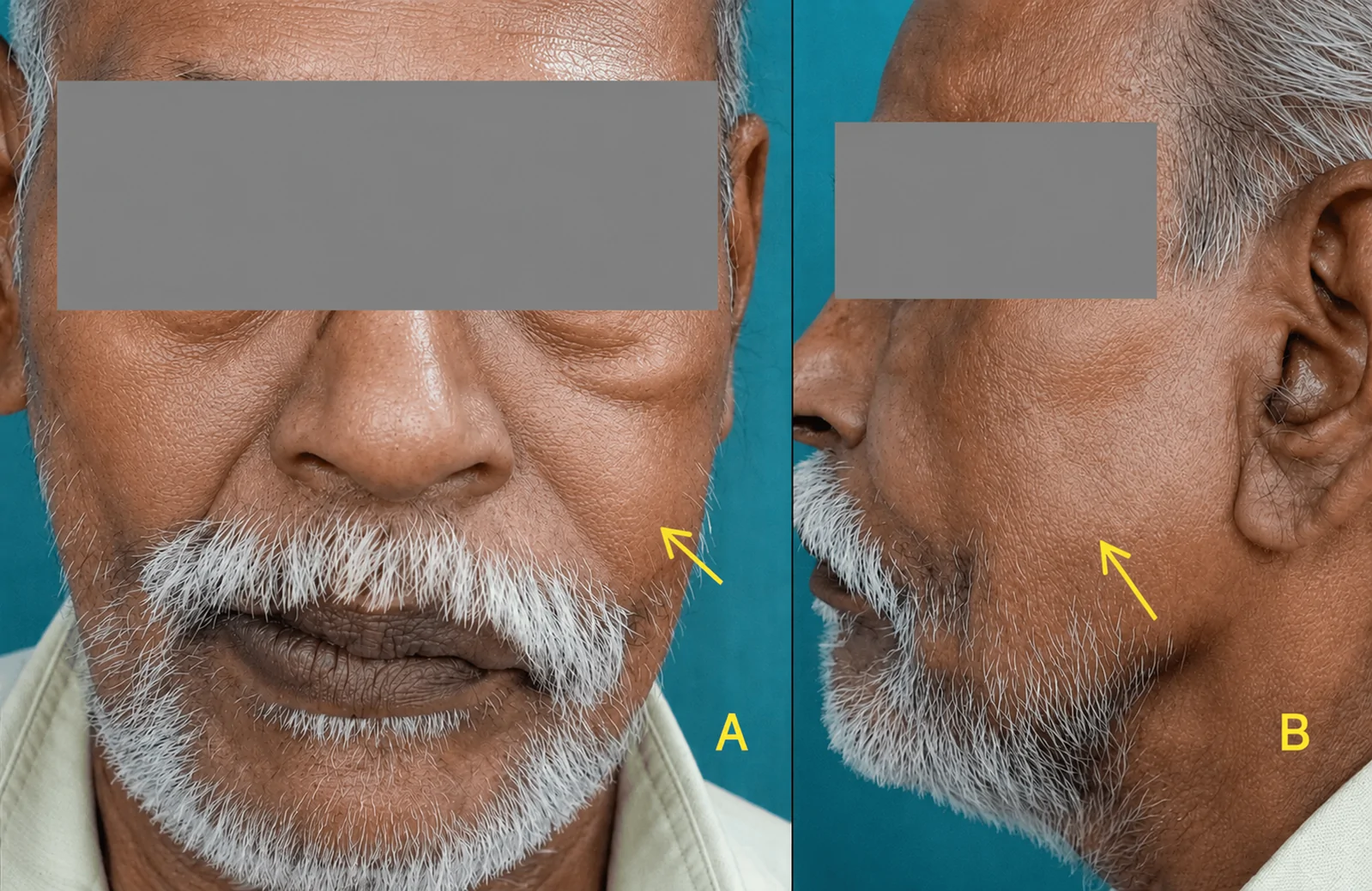
Case two frontal and left lateral profile views A) Extraoral swelling on the left side of the face (yellow arrow indicates the swelling). B) Extraoral swelling on the left cheek (yellow arrow indicates the swelling).

A 57-year-old male reported to our department with a chief complaint of swelling and pain involving the left side of the upper lip and the middle third of the face for the past two months. The patient gave a history of tooth pain in the decayed left upper back teeth region and visited a private dental practitioner, who prescribed metronidazole 400 mg tablets thrice a day, a capsule of Amoxicillin 500 mg thrice a day, and an analgesic tablet of aceclofenac 100 mg for five days thrice a day for his tooth pain. The swelling persisted despite the intake of a course of antibiotics. The patient continued taking antibiotics intermittently for the next week, but the swelling persisted. On extraoral clinical examination, facial asymmetry was present due to swelling on the left side of the upper lip and the middle third of the face. A swelling measuring around 4 cm × 3 cm was present at the left middle third of the face, extending 1 cm below the left lower eyelid, anteriorly 2.5 cm away from the left ala of the nose, and posteriorly the swelling extended 1 cm in front of the left preauricular region. The skin over the swelling appeared stretched with no secondary changes, such as sinus, ulcer, or punctum (Figures 5A, 5B).

**Figure 6:**
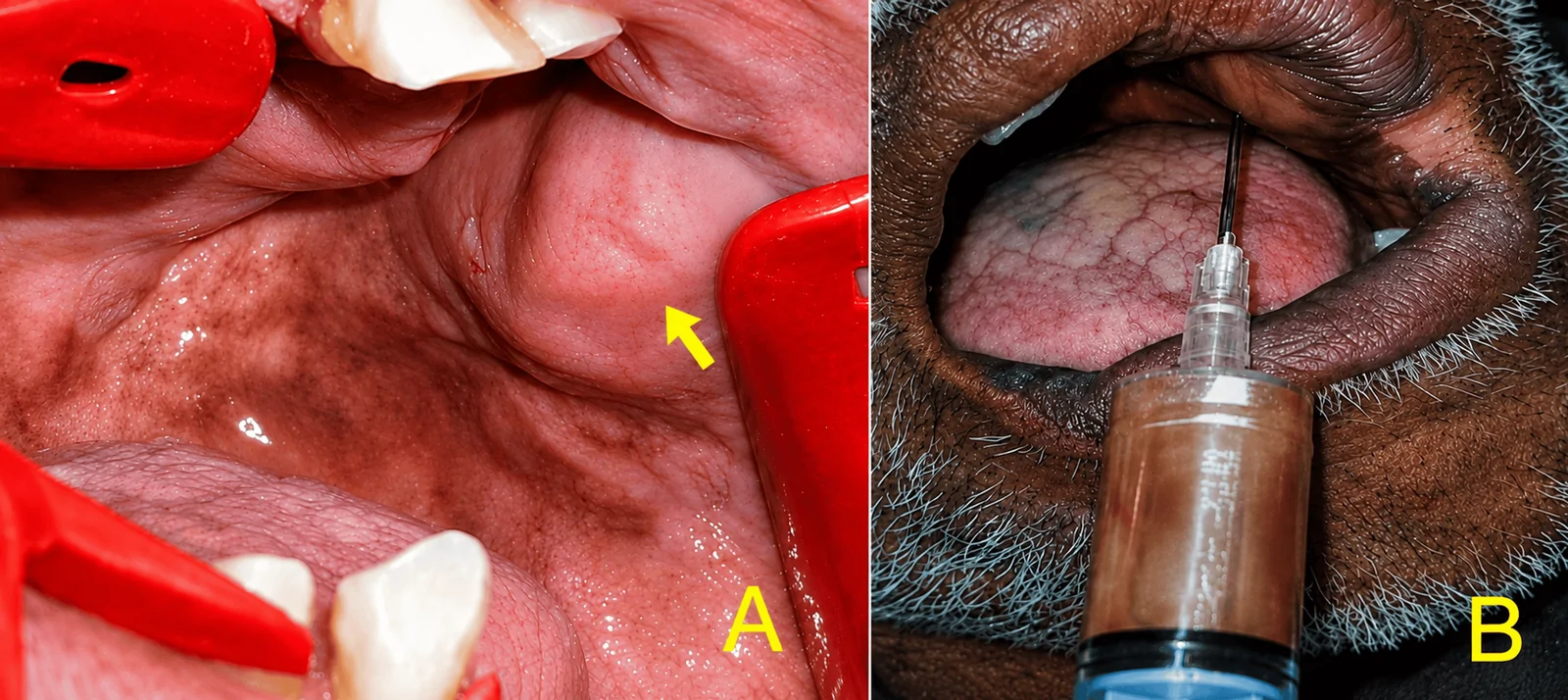
Case two intraoral examination A) Intraoral swelling on the left buccal mucosa (yellow arrow indicates the swelling), B) Aspiration revealed yellow coloured discharge mixed with blood.

On palpation, the swelling was non-tender and firm in consistency. Intraoral examination revealed carious root stumps 24 and 25 and also an intraoral swelling involving the left buccal mucosa (Figure 6A). Aspiration revealed yellow coloured discharge mixed with blood (Figure 6B).

**Figure 7:**
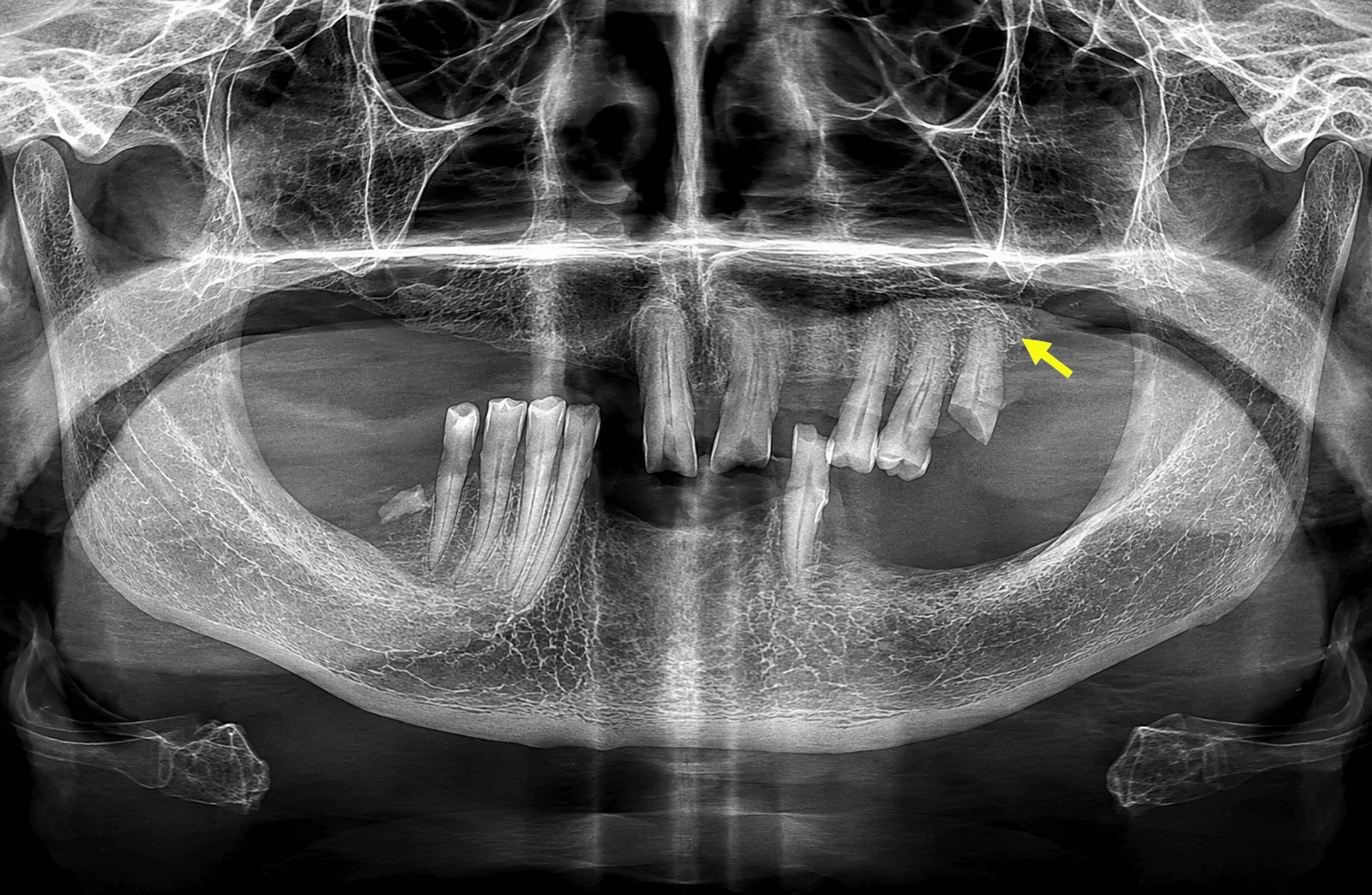
Case two - orthopantomography revealed a carious root stump in the left maxillary second premolar (yellow arrow indicates the infected carious tooth)

The orthopantomography revealed a carious root stump in the left maxillary second premolar (Figure 7).

The hematological laboratory parameters assessed are tabulated (Table 2).

**Table 2:** Case two - hematological laboratory parameters

Hematological Laboratory Parameter	Observed value	Normal Value
Random Blood Glucose	114 mg/ dL	80-140 mg/dL
Bleeding time	4 minutes	2- 7 minutes
Clotting time	4 minutes	8-15 minutes
Total Leukocyte count	8400 cells / cubic mm	5000-10,000 cells / cubic mm
Neutrophil count	58%	40-70%
Eosinophil count	2%	1-2%
Lymphocyte count	40 %	20-40%
Erythrocyte Sedimentation Rate (ESR)	1 hour = 24 mm	1 hour = 20 mm

In both the above cases, based on history and clinical findings, a provisional diagnosis of orofacial antibioma was made. The differential diagnosis of orofacial antibioma includes dentoalveolar abscess, buccal space infection, and canine space infection. In canine space infection, characteristic obliteration of the nasolabial fold is present. Prominent cheek swelling occurs in both buccal space infection and orofacial antibioma. The swelling caused by space infection is usually doughy, woody, or brawny in consistency. The swelling caused by a dentoalveolar abscess is usually soft and fluctuant due to pus on palpation, associated with fever. The progression of the swelling in a dentoalveolar abscess or space infection begins to slow down or resolve on antibiotic therapy, whereas in antibioma, the swelling remains completely unchanged or hardens further despite antibiotic therapy.

In contrast, orofacial antibioma is associated with swelling that is firm, tough, and indurated, with or without minimal systemic signs of inflammation, such as fever. Microscopy and culture reveal pyogenic microorganisms in abscess and space infections. In antibioma, the abscess is usually sterile, and microscopy and culture are negative. Extraction of the carious right maxillary second molar tooth under a posterior superior alveolar nerve block and extraction of the carious root stump in the left maxillary second premolar were performed under a middle-superior alveolar nerve block using 2% lignocaine local anaesthesia. Follow-up evaluation after a week revealed satisfactory healing in both cases.

## Discussion

Antibioma is an uncommon clinical entity characterized by the formation of a localized, relatively well-circumscribed inflammatory mass following inadequate drainage of an infective focus in the setting of prolonged or inappropriate antibiotic therapy [1]. Although the term has been described in association with infections at several anatomical sites, its occurrence in the maxillofacial region is uncommon and may create a diagnostic challenge because the lesion can clinically resemble a persistent odontogenic infection, neoplasm, granulomatous lesion, or other chronic inflammatory process. The underlying concept is that antimicrobial therapy may suppress the acute inflammatory manifestations of an infection without eliminating the focus of infection, particularly when a closed collection has not been adequately drained. In such circumstances, organization of the inflammatory exudate and fibrosis may result in a firm, persistent mass.

The molecular pathogenesis of antibioma is not yet defined by a single antibioma-specific signaling pathway; rather, its formation can be understood as the consequence of persistent microbial stimulation, antibiotic-tolerant bacterial populations, chronic inflammation, and progressive fibrotic organization of an inadequately drained infectious focus. Following antibiotic exposure, susceptible planktonic bacteria are reduced, whereas bacteria within biofilms or metabolically quiescent persister populations may survive because extracellular polymeric substances, altered metabolic states, restricted antimicrobial penetration, and bacterial stress-response pathways confer substantial antibiotic tolerance. Recent evidence indicates that biofilm-associated persistence is mediated by complex bacterial regulatory mechanisms, including altered metabolism, extracellular matrix production, and toxin-antitoxin systems [1-3]. Persistent bacteria and their microbial products continue to stimulate innate immune pathways, maintaining macrophage and neutrophil activation and the release of inflammatory mediators. Chronic macrophage activation subsequently promotes a profibrotic microenvironment characterized by transforming growth factor (TGF-β), platelet derived growth factor (PDGF), and other cytokines, which activate fibroblasts and promote myofibroblast differentiation and extracellular-matrix deposition through canonical TGF-β/ SMA-from c.elegans worm gene "Small", MAD - from the drosophila fly gene "Mothers Against Decapentaplegic" (SMAD) and related signaling pathways [2,3]. Recent experimental evidence from Staphylococcus aureus abscesses has additionally demonstrated that macrophage-derived amphiregulin can activate Epidermal Growth Factor Receptor- m Mammalian Target of Rapamycin/ yes-associated protein (EGFR-mTOR/YAP signaling in surrounding precursor cells, promoting myofibroblast differentiation, vascular constriction, impaired local perfusion, and reduced antibiotic delivery, thereby facilitating bacterial persistence within an abscess [4]. Collectively, these mechanisms provide a plausible molecular basis for the development of antibioma, in which incomplete microbial eradication following antibiotic therapy permits persistence of biofilm-associated or antibiotic-tolerant organisms, sustains innate immune activation, promotes macrophage-mediated profibrotic signaling, and ultimately results in myofibroblast activation, collagen deposition, and progressive fibrous encapsulation of the residual inflammatory focus [4]. Importantly, this mechanism should be regarded as a proposed model extrapolated from contemporary studies of abscess persistence, biofilm biology, and fibrosis rather than as a molecular pathway uniquely demonstrated in antibioma.

Odontogenic infections are predominantly polymicrobial and commonly involve both aerobic and anaerobic microorganisms. Successful management therefore depends primarily on elimination of the source and establishment of adequate drainage or decompression rather than on antimicrobial therapy alone [5]. Studies of odontogenic and maxillofacial space infections have demonstrated that surgical drainage, removal of the etiological focus, and appropriate antimicrobial therapy when indicated are the major components of successful treatment [5,6]. Antibiotics have limited penetration into a walled-off purulent collection, and consequently, their indiscriminate use in the absence of adequate source control may result in temporary symptomatic improvement without resolution of the underlying pathology.

The development of an antibioma is particularly relevant when considering the inappropriate prescription of antibiotics. A systematic review of the antibiotic therapy of odontogenic infections demonstrated that, after adequate drainage and removal of the source of infection, different antibiotic regimens generally resulted in similar clinical outcomes, highlighting the value of local intervention over antibiotic choice [5]. Similarly, current evidence-based guidelines stress that antibiotics should not be considered a replacement for definitive dental treatment. For localized odontogenic infections without systemic involvement, definitive treatment such as drainage, endodontic therapy, or extraction should be prioritized, whereas systemic antimicrobial therapy is principally indicated when there is systemic involvement or a significant risk of progression [5].

The clinical presentation of antibioma may therefore be misleading. As opposed to an acute odontogenic abscess, which typically presents with pain, tenderness, erythema, fluctuance and other signs of active inflammation, an antibioma may present as a relatively firm, indurated, slowly enlarging or persistent swelling with minimal systemic manifestations. This change in clinical picture may be due to partial suppression of the original infection by antimicrobial therapy and subsequent organization of the lesion. Consequently, a persistent swelling despite an apparently adequate course of antibiotics should prompt reconsideration of the diagnosis rather than repeated empirical antimicrobial treatment [5].

Imaging plays an important role in this diagnostic process. Conventional radiography can identify a possible odontogenic source or osseous involvement, whereas ultrasonography, computed tomography, or magnetic resonance imaging may provide additional information regarding the nature, extent, and anatomical relationships of a soft-tissue lesion. Importantly, imaging findings should be interpreted together with the clinical history, particularly the duration and type of previous antibiotic therapy. In a patient with a history of repeated antibiotic administration, persistent lesion should raise suspicion of an organized collection or chronic inflammatory lesion rather than an unresolved acute abscess [5].

Another important consideration is the possibility of a culture that is sterile. Prior antimicrobial exposure can reduce the microbiological yield from an infected collection, and therefore a negative culture does not necessarily exclude a previous or partially treated infection. This is particularly relevant in lesions that have been subjected to prolonged antimicrobial therapy. Histopathological examination may demonstrate granulation tissue, fibrosis, chronic inflammatory cell infiltration, and, in some cases, foreign-body-type inflammatory changes, thereby supporting the diagnosis when clinical and microbiological findings are inconclusive [5].

The management of antibioma should be directed toward definitive source control. Depending upon the anatomic location and the basic etiology, treatment may consist of incision and drainage, complete excision of the organized lesion, removal of the odontogenic source, or a combination of these. Instead of continuing empirically for long periods of time, antibiotics should be chosen based on clinical severity and microbiological findings, where available. In localized infection without systemic involvement, definitive dental therapy and drainage can be performed promptly, and routine antibiotic therapy may provide little additional benefit [5]. Conversely, systemic manifestations, progressive infection, involvement of deep anatomical spaces or the presence of significant risk factors for complications require adequate antimicrobial therapy and definitive intervention [5,6].

The case also points out the necessity of antimicrobial stewardship in dental practice. Overuse or long-term use of antibiotics can cause adverse drug reactions, lead to resistant organisms and other complications related to antibiotics. There is international agreement that dental antibiotic stewardship needs appropriate prescribing and standardized outcomes for antimicrobial stewardship in dentistry [6]. This issue is especially relevant in India, where a recent review of dental policy has pointed out the lack of dental-specific antibiotic prescribing guidance and stewardship resources [7]. Clinicians should therefore avoid using antibiotics as a substitute for definitive management of localized dental infection and should educate patients that antimicrobial therapy alone cannot reliably resolve a closed collection.

From a diagnostic perspective, antibioma should be considered when a patient presents with a persistent or recurrent maxillofacial swelling following repeated courses of antibiotics, particularly when the clinical response is incomplete or the lesion becomes firm and relatively asymptomatic. The differential diagnosis should include residual odontogenic infection, chronic osteomyelitis, inflammatory or reactive lesions, granulomatous disease, benign and malignant neoplasms, and foreign-body reactions. A detailed history of previous antimicrobial exposure, appropriate imaging, microbiological evaluation when feasible, and histopathological examination of excised tissue are useful in establishing the diagnosis and excluding clinically significant mimics.

The principal clinical lesson is that antibiotics should complement, rather than replace, definitive treatment of odontogenic infection. The formation of an antibioma illustrates the potential consequence of suppressing an infective process without adequately eliminating its source. Contemporary evidence supports a shift from empirical, prolonged antibiotic prescribing toward early diagnosis, effective drainage or source control, targeted antimicrobial therapy when indicated, and close clinical reassessment [6-8]. Recognition of this uncommon entity can prevent repeated ineffective antibiotic courses and facilitate timely definitive management.

Diagnosing orofacial antibioma remains challenging, as the clinical features may overlap with other orofacial infections, necessitating a comprehensive approach to differential diagnosis. Careful history taking, clinical examination, and appropriate diagnostic tests, such as ultrasonographic imaging and microbiological analysis, are crucial in establishing a definitive diagnosis and guiding appropriate treatment [6,7].

Managing orofacial antibioma requires a multidisciplinary approach involving antimicrobial therapy, surgical intervention, and supportive care. The specific microbial pathogens involved guide the selection of appropriate antimicrobial agents. At the same time, surgical interventions, such as incision and drainage, may be necessary to address localized abscesses or areas of tissue necrosis. Orofacial antibiomas can be managed using a periodic dressing of magnesium sulfate and glycerin.

The recognition of the potential for serious complications of orofacial antibiomas, such as intracranial extension into the brain, pleuropulmonary involvement with suppurative mediastinal lung infections, retropharyngeal spread, airway obstruction, and hematogenous dissemination of microorganisms resulting in septicemia. These complications of orofacial antibiomas highlight the need for early and aggressive intervention to prevent such adverse outcomes [8-15]. Orbital antibioma can occur as a sequela of inadequate treatment of recurrent rhino-nasal sinusitis [16]. 

Antibioma may manifest as a resistant wound, especially after surgery for an infection. In painless circumstances, diagnosis is challenging. Successful wound healing depends on monitoring for antibiomas, mainly when there is inadequate drainage or potential antibiotic overuse. After early discovery through careful examination, successful treatment can be achieved with surgery and the appropriate medications [16]. The proper antibioma diagnosis is always reached in correlation with history [17].Baheti A, Sankhe S, and Mahore A reported a case of antibioma involving the common peroneal nerve, resulting in neuralgic pain following surgical removal of osteochondroma, which developed in the right fibular neck. No organisms are found by routine microscopy and culture. Magnetic resonance imaging (MRI) of antibioma reveal a hyperintense area with peripheral postcontrast enhancement in T2 image,characteristics are typical of a classic chronic abscess [18].

Clinical significance

This case series emphasizes the necessity of a detailed clinical history, particularly previous antibiotic exposure, in the diagnosis of orofacial antibioma. In patients who have had multiple courses of antibiotics and have persistent or recurrent orofacial swelling, clinicians should consider an organized chronic inflammatory lesion and reassess the source of infection rather than continuing empirical antibiotics. The clinical presentation is variable, may simulate other inflammatory, infectious or neoplastic lesions, making the diagnosis difficult. Early diagnosis and definitive management can be achieved with awareness of the unique history and clinical features, along with appropriate imaging and histopathological evaluation where indicated. These cases also emphasize the necessity of antibiotic stewardship and definitive control of the underlying odontogenic or infectious source to prevent unnecessary or prolonged antimicrobial therapy.

## Conclusions

Antibioma is an uncommon but clinically important complication of inadequate or prolonged antibiotic therapy following an infectious process. It can manifest as a persistent, firm, and sometimes painless mass mimicking neoplastic or other chronic inflammatory lesions, posing a diagnostic challenge. A detailed history of prior infection, antibiotic use, physical examination, and appropriate imaging may help establish the diagnosis and avoid unnecessary invasive procedures. The recognition of antibiomas is especially important in the oral and maxillofacial region, in which chronic post-infectious lesions may mimic benign or malignant neoplasms. A high index of suspicion should be kept while evaluating a persistent mass at a previously infected site, especially in patients with a history of incomplete or prolonged antimicrobial treatment. The diagnosis of antibioma in these cases remains presumptive based primarily on clinical history and ultrasound findings. However, clinical follow-up, appropriate antimicrobial management, and drainage or removal of the underlying source of infection when indicated resulted in resolution of these lesions. Early recognition of an antibioma may result in appropriate treatment and prevent unnecessary diagnostic and surgical procedures.
